# Neutrophil-to-Lymphocyte Ratio as a Predictor of Response to Peginterferon plus Ribavirin Therapy for Chronic Hepatitis C

**DOI:** 10.1155/2014/462958

**Published:** 2014-11-18

**Authors:** Ming-Te Kuo, Tsung-Hui Hu, Sheng-Nan Lu, Chao Hung Hung, Jing-Houng Wang, Chien-Hung Chen, Yi-Chun Chiu, Chuan-Mo Lee

**Affiliations:** Division of Hepato-Gastroenterology, Department of Internal Medicine, Kaohsiung Chang Gung Memorial Hospital and Chang Gung University College of Medicine, 123 Ta Pei Road, Niao Sung, Kaohsiung 833, Taiwan

## Abstract

We aimed to determine whether neutrophil-to-lymphocyte ratio (NLR) could be a predictor of antiviral response in chronic hepatitis C patients. A total of 602 consecutive patients (genotype 1, *n* = 263; genotype 2, *n* = 297; others/unknown, *n* = 42) receiving response-guided therapy with peginterferon plus ribavirin were recruited. NLR was related to clinical and virological features and to treatment outcome. Rapid virological response (RVR) and sustained virological response (SVR) were achieved in 436 (73%) and 458 (76%) of the patients, respectively. Higher NLR (≥1.42) was found to be associated with higher prevalence of DM (*P* = 0.039) and higher hepatitis C viral load (*P* = 0.002) and white cell count (*P* < 0.001). NLR was significantly lower in patients with RVR and SVR compared to those without (*P* = 0.032 and 0.034, resp.). However, NLR was not an independent factor by multivariate analysis. In the subgroup analysis, higher NLR (≥1.42) (odds ratio, 0.494, *P* = 0.038) was an independent poor predictor of SVR in genotype 2 patients but was not in genotype 1 patients. In conclusion, NLR is a simple and easily accessible marker to predict response to peginterferon plus ribavirin therapy for chronic hepatitis C genotype 2.

## 1. Introduction

Chronic hepatitis C virus (HCV) infection can lead to chronic hepatitis, liver cirrhosis, and eventually hepatocellular carcinoma (HCC) [1–3]. The associated complications, mortality, and need for liver transplantation are worldwide problems [3]. The treatment goal of chronic hepatitis C is to achieve sustained virological response (SVR), which can decrease remarkably the associated complications of end stage liver disease and the risk of HCC development [4–6]. Nowadays, the optimal treatment regimen for chronic HCV infection is unclear since many new direct antiviral agents have been developing [7, 8]. Peginterferon plus ribavirin remains the current first line of therapy for HCV in resource-limited settings where these new therapies cannot be afforded [9, 10]. Therefore, it is of clinical importance to identify patients who are or are not good candidates for peginterferon plus ribavirin therapy. Several factors have been reported to predict the treatment response of peginterferon plus ribavirin therapy, including baseline viral loads [11], HCV variations [12], race, interleukin (IL)28B polymorphisms [12, 13], age, body weight [14], insulin resistance [15], and so forth.

Neutrophil-to-lymphocyte ratio (NLR) is a novel-potential laboratory marker to determine systemic inflammation in the body and being measured routinely in peripheral blood. This ratio can be obtained easily from the differential white blood cell (WBC) count. It has a greater predictability than total WBC count or neutrophil count as a useful prognostic marker in cardiovascular diseases [16]. It has been reported to be associated with adverse outcome in various types of cancer, including colorectal cancer [17], esophageal cancer [18], gastric cancer [19], and pancreatic cancer [20]. In addition, recent data have also suggested that an elevated NLR may correlate with worse prognosis in patients with HCC who underwent transcatheter arterial chemoembolization, radiofrequency, resection, or orthotopic liver transplantation [21–24].

To our knowledge, NLR and the association of clinical features and antiviral response in chronic hepatitis C patients have not been investigated. Thus, we conducted a large cohort of chronic hepatitis C patients receiving response-guided therapy with peginterferon plus ribavirin to clarify these issues.

## 2. Materials and Methods

### 2.1. Patients

From January 2010 to October 2012, we enrolled 602 naïve patients with chronic HCV infection who were eligibly treated with peginterferon and ribavirin combination therapy in single medical center. The diagnosis of chronic hepatitis C was seropositive for HCV antibodies and detectable HCV RNA for more than 6 months. Clinical diagnosis of cirrhosis was based on repeated ultrasound findings suggestive of cirrhosis at least twice 3 months apart, supplemented with clinical criteria or other signs of portal hypertension [25]. Patients were excluded if they were positive for serum hepatitis B surface antigen or anti-human immunodeficiency virus antibody or exhibited other causes of hepatocellular injury (alcoholism, autoimmune liver disease, or treatment with hepatotoxic drugs). In addition, patients with uncontrolled diabetes, heart failure, coronary artery diseases, arrhythmia, chronic systemic inflammatory disease, malignancy, and other diseases which might affect the NLR were also excluded.

Patients were treated according to the on-treatment response as follows: 24 weeks for patients achieving a rapid virological response (RVR, seronegativity of HCV RNA at 4 weeks of therapy), 48 weeks for those with an early virological response (EVR, at least a 2-log10 decrease from baseline of serum HCV RNA at 12 weeks of treatment) but no RVR, and early termination (<16 weeks) in those without an EVR [12]. This protocol was recommended by the National Health Insurance Bureau in Taiwan since November 2009. All patients received either peginterferon alfa-2a (180 *μ*g/week) or peginterferon alfa-2b (1.5 *μ*g/kg/week) subcutaneously plus weight-based ribavirin (1000 mg/d for weight < 75 kg and 1200 mg/d for weight > 75 kg). SVR was defined as undetectable HCV RNA throughout 24 weeks of posttreatment follow-up period. This study protocol conformed to the ethical guidelines of the 1975 Declaration of Helsinki and was approved by the ethical committees of Chang Gung Memorial Hospital.

### 2.2. Laboratory Assays

Before treatment, qualitative detection of HCV RNA was performed by a standardized qualitative reverse transcription- (RT-) PCR assay (Amplicor, Roche Diagnostics, Branchburg, NJ, USA), using biotinylated primers for the 5′ noncoding region. The lowest detection limit of this assay was 50 international units (IU)/mL. Serum HCV RNA levels were determined by COBAS TaqMan HCV Test (TaqMan HCV; Roche Molecular Systems Inc., Branchburg, NJ, lower limit of detection: 15 IU/mL). Genotyping of HCV was performed by reverse hybridization assay (Inno-LiPA HCV II; Innogenetics N.V., Gent, Belgium) using the HCV-Amplicor products.

NLR was calculated from the differential count by dividing the neutrophil measurement by the lymphocyte measurement before treatment. None of the patients showed the clinical signs of acute infection or stress or received medications affecting the number of leukocytes. Laboratory data, including aspartate aminotransferase (AST), alanine aminotransferase (ALT), platelet counts, baseline viral load, and IL28B polymorphism were also collected.

### 2.3. Statistical Analysis

Continuous data are expressed as mean ± standard deviation, and the categorical data are expressed as number (percentage). Comparisons of differences in categorical date between groups were performed using the chi-square test. Distributions of continuous variables were analyzed by Student's *t*-test or Mann-Whitney *U* test for the two groups where appropriate. The best cutoff point of NLR for predicting SVR was determined by receiver operating characteristic curve analysis. Stepwise logistic regression analysis was used to identify the independent factors associated with SVR. A *P* value of less than 0.05 was considered significant.

## 3. Results

### 3.1. Patient Baseline Characteristics

Patient characteristics are shown in Table 1. They were 316 men and 286 women, 21 to 81 years old, with a mean age of 54.3 ± 11.1 years. Of them, 91 patients (15%) had cirrhosis and 103 (17%) patient had diabetes. Two hundred sixty-three (45%) patients were infected with genotype 1 and 297 (50%) were infected with genotype 2. The pretreatment mean NLR was 1.6.

Of these patients, 436 (72.5%) patients achieved RVR, 152 (25.2%) achieved an EVR without RVR, and 14 (2.3%) had no EVR. The rates of SVR were 85% of RVR patients, 58% of EVR patients, and 0% of non-EVR patients, respectively.

### 3.2. Analysis of Factors Associated with RVR and SVR

As shown in Table 2, significant factors associated RVR were young age (*P* = 0.032), IL28B CC genotype (*P* < 0.001), non-cirrhosis (*P* < 0.001), low viral load (*P* < 0.001), HCV genotype non-1 (*P* < 0.001), and lower NLR (*P* = 0.032) by univariate analysis. On the other hand, young age (*P* = 0.006), IL28B CC genotype (*P* = 0.004), non-cirrhosis (*P* < 0.001), low viral load (*P* < 0.001), HCV genotype non-1 (*P* < 0.001), RVR achievement (*P* < 0.001), lower NLR (*P* = 0.034), and high platelet count (*P* = 0.037) were significantly associated with SVR by univariate analysis.

### 3.3. Comparison of Chronic Hepatitis C Patients with Higher or Lower NLR

**Figure 1:**
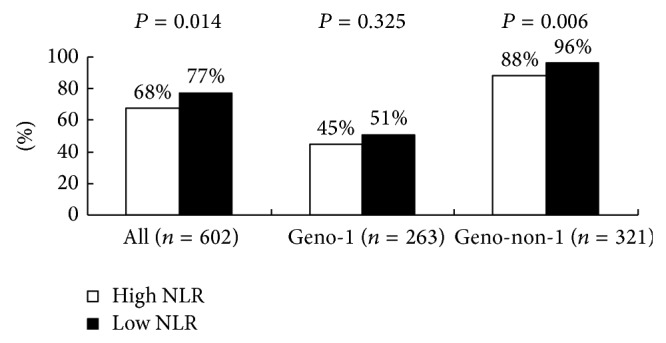
The comparison of RVR between patients with lower NLR (<1.42) and higher NLR (≥1.42), as determined by using ROC curve analysis.

**Figure 2:**
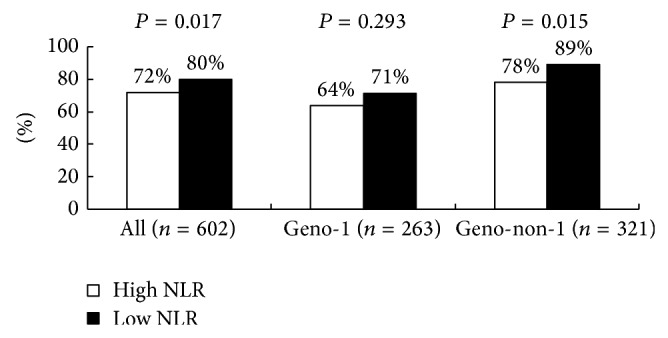
The comparison of SVR between patients with lower NLR (<1.42) and higher NLR (≥1.42).

Figures 1 and 2 show the comparison of RVR and SVR rate between patients with lower NLR (<1.42) and higher NLR (≥1.42), as determined by using ROC curve analysis. In HCV genotype non-1 group, patients with higher NLR had a lower rate of RVR and SVR compared to those with lower NLR (RVR: *P* = 0.006, SVR: *P* = 0.015, resp.), while in HCV geno-1 group, these significant differences were not found.

Table 3 shows the comparison of clinical and laboratory characteristics of chronic hepatitis C patients with higher or lower NLR. We found that patients with higher NLR had higher prevalence of DM (*P* = 0.039) and higher HCV load (*P* = 0.002) and white cell count (*P* < 0.001) as compared to those with lower NLR. These significant associations were not different between genotype 1 and non-1 patients.

### 3.4. Multivariate Analysis of Factors Associated with SVR

Based on stepwise logistic regression analysis, the achievement of RVR (odds ratio (OR): 2.293, 95% confidence interval (CI): 1.325–3.970; *P* = 0.003), IL28B CC genotype (OR: 2.482, 95% CI: 1.332–4.627; *P* = 0.004), low viral load (OR: 2.227, 95% CI: 1.342–3.690; *P* = 0.002), and high platelet counts (OR: 1.695, 95% CI: 1.054–2.723; *P* = 0.029) were independent factors predicting SVR in this cohort. As for genotype 1 patients, only RVR (OR: 2.786, 95% CI: 1.487–3.970; *P* = 0.001) and IL28B CC type (OR: 2.881, 95% CI: 1.216–6.827; *P* = 0.016) were independent factors, while among genotype 2 patients, high NLR (OR: 0.494, 95% CI: 0.253–0.963; *P* = 0.038) was an independent factor predicting SVR, in addition to low viral load (OR: 3.086, 95% CI: 1.570–6.060; *P* = 0.001), RVR achievement (OR: 2.873, 95% CI: 1.113–7.19; *P* = 0.029), and high platelet counts (OR: 2.417, 95% CI: 1.240–4.714; *P* = 0.01) (Table 4).

## 4. Discussion

Over the past decade, several factors probably affecting the treatment response to peginterferon plus ribavirin therapy for chronic hepatitis C have been reported [11–15]. Of great importance, IL28B polymorphism and viral factors including genotype, viral load, and HCV variations have been the strongest predictors [11–13]. In this present study, we investigated the role of NLR, an emerging parameter that reflects systemic inflammation as well as the general nutrition status, in the association of antiviral response for chronic hepatitis C. Although univariate analysis showed the significant association of NLR with RVR and SVR, NLR was not an independent factor by stepwise logistic regression analysis, while in the subgroup analysis, higher NLR was an independent poor predictor of SVR in patients with genotype 2 but was not in those with genotype 1. To our knowledge, this is the first report discussing the utility of NLR to predict treatment response to peginterferon plus ribavirin therapy for chronic hepatitis C. However, further studies are necessary to understand the different impact of NLR on antiviral response between genotypes.

In our study, we have found a significant correlation between NLR and HCV load. This finding could partially explain the negative impact of higher NLR on the antiviral response. Although the precise mechanism in this association remains unclear, it can be speculated that higher NLR partially from the reduction of lymphocyte count is involved in the enhanced HCV replication. This hypothesis is supported by a recent study showing that lymphopenia was significantly associated with HCV replication and higher rates of HCV recurrence after liver transplantation [26]. On the other hand, another interesting finding of our study was that higher NLR was associated with higher prevalence of DM. This finding was consistent with recent evidence that showed a significant positive correlation of NLR with the development of insulin resistance and type 2 DM [27]. Given the association between insulin resistance/DM and poor response to IFN-based therapy [15], higher NLR might therefore affect the treatment outcome in chronic hepatitis C patients although there was a borderline significance between DM and SVR in our cohort. Further studies are necessary to clarify the relationship between insulin resistance and NLR.

Previous studies have discussed the association of differential WBC count and response to peginterferon plus ribavirin therapy in chronic hepatitis C. A higher baseline neutrophils count (>2300/uL) was reported to predict a greater likelihood of SVR [28]. Another study showed that lymphocytosis was associated with lower rate of SVR [29]. These findings were considerably different from that in our study. However, these contradictory results could be explained by the smaller case numbers in their studies and the limited interpretation since absolute number of individual cell types is more easily affected by different situation, including infection, stress, or medication and various physiological conditions such as dehydration and exercise [16]. In contrast, NLR is a ratio of two different complementary immune pathways, thus integrating the deleterious effects of neutrophils. Using NLR instead of either one cell count level should be more objective.

Currently, there is no universal cut-off point of NLR for predicting clinical outcomes in a variety of diseases. As the prognostic role in patients with cancer, a threshold of NLR > 5 was the most consistently used. In patients with cancer, neutrophil levels can modify and provide an adequate environment for tumor progression and development [30]. Increasing infiltration of CD4+ T lymphocytes at tumor margins has been proven to be associated with less recurrence and better prognosis in colorectal cancer and HCC [31, 32]. It is also well established that cancer-related systemic inflammatory response is associated with alternation in circulating white blood cells, specifically the presence of neutrophilia with a relative lymphocytopenia [33]. Taken together, these factors could contribute to the higher threshold of NLR in cancer patients, while in patients with chronic hepatitis C, the best cutoff point of NLR for predicting SVR was relatively lower (1.42 in our cohort). This might be related to virus infection that could lead to elevated lymphocyte level and lower threshold of NLR.

## 5. Conclusion

Our study demonstrated that higher NLR was associated with higher prevalence of DM and higher hepatitis C viral load. NLR is a simple and easily accessible marker to predict treatment response to peginterferon plus ribavirin therapy for chronic hepatitis C genotype 2 patients. Further studies are needed to externally cross-validate our finding in other cohorts.

## Figures and Tables

**Table 1:** Baseline characteristics of the study population.

Variables	
Age (mean ± SD) (yr)	54.3 ± 11.1
Gender (male/female)	316/286
rs12979860 (CC/CT or TT)^*^	422/67
Cirrhosis (%)	91 (15%)
DM (%)	103 (17%)
BMI (kg/m^2^)	24.7 ± 3.7
Log HCV-RNA (IU/mL)	5.4 ± 1.0
Genotype 1/2/3/5/6^**^	263/297/9/1/14
WBC (10^3^/*μ*L)	5.8 ± 1.7
N-L ratio	1.60 ± 0.89
Platelet (10^4^/*μ*L)	17.6 ± 5.9

Available in 489 (81%)^*^ and 584 (97%)^**^ of patients.

DM: diabetes mellitus; BMI: body mass index; RVR: rapid virological response; WBC: white cell count; N-L ratio: neutrophil-lymphocyte ratio.

**Table 2:** Factors associated with rapid virological response and sustained virological response to peginterferon plus ribavirin in chronic hepatitis C patients.

	RVR (*n* = 436)	Non-RVR (*n* = 166)	*P* value	SVR (*n* = 458)	Non-SVR (*n* = 144)	*P* value
Age (mean ± SD) (yr)	53.7 ± 11.5	55.8 ± 10.0	0.032	53.6 ± 11.1	56.5 ± 10.8	0.006
Gender (male/female)	237/199	79/87	0.082	243/215	73/71	0.634
rs12979860 (CC/CT or TT)	312/34	110/33	<0.001	326/40	96/27	0.004
Cirrhosis (%)	57 (13%)	34 (20%)	<0.001	51 (11%)	40 (28%)	<0.001
DM (%)	76 (17%)	27 (16%)	0.418	71 (16%)	32 (22%)	0.075
BMI (kg/m^2^)	24.6 ± 3.7	25.1 ± 3.9	0.161	24.6 ± 3.6	25.1 ± 4.1	0.161
Log HCV-RNA (IU/mL)	5.2 ± 1.0	6.1 ± 0.6	<0.001	5.3 ± 1.0	5.9 ± 0.7	<0.001
Genotype 1/non-1	126/296	137/25	<0.001	177/269	86/52	<0.001
RVR (%)	—	—	—	370 (81%)	66 (46%)	<0.001
WBC (10^3^/*μ*L)	5.8 ± 1.6	5.8 ± 1.9	0.597	5.8 ± 1.7	6.0 ± 4.2	0.482
N-L ratio	1.55 ± 0.82	1.74 ± 1.05	0.032	1.55 ± 0.75	1.78 ± 1.24	0.034
Platelet (10^4^/*μ*L)	17.7 ± 5.7	17.2 ± 6.4	0.424	17.9 ± 5.6	16.6 ± 6.8	0.037

RVR: rapid virological response; DM: diabetes mellitus; BMI: body mass index; HCV: hepatitis C virus; RVR: rapid virological response; N-L ratio: neutrophil-lymphocyte ratio.

**Table 3:** Comparison of clinical and laboratory characteristics between high and low neutrophil-lymphocyte ratio of chronic hepatitis C patients.

	High N-L ratio ≥ 1.42^*^ (*n* = 306)	Low N-L ratio < 1.42 (*n* = 296)	*P* value
Age (mean ± SD) (yr)	53.5 ± 11.8	55.0 ± 10.4	0.101
Gender (male/female)	169/137	147/149	0.192
rs12979860 (CC/CT or TT)	218/32	96/27	0.600
Cirrhosis (%)	53 (17%)	38 (13%)	0.139
DM (%)	61 (20%)	42 (14%)	0.039
BMI (kg/m^2^)	24.6 ± 4.0	24.9 ± 3.4	0.299
Log HCV-RNA (IU/mL)	5.6 ± 0.9	5.3 ± 1.0	0.002
Genotype 1/non-1	140/158	123/163	0.360
WBC (10^3^/*μ*L)	6.2 ± 3.2	5.4 ± 1.5	<0.001
Hemoglobin (g/dL)	14.1 ± 1.7	14.1 ± 1.4	0.846
Platelet (10^4^/*μ*L)	17.8 ± 6.1	17.3 ± 5.7	0.307

^*^As determined by ROC curve for best cut-off for predicting SVR.

N-L ratio: neutrophil-lymphocyte ratio; DM: diabetes mellitus; BMI: body mass index; RVR: rapid virological response; WBC: white cell count.

**Table 4:** Stepwise logistic regression analysis of factors associated with sustained virological response.

	Odds ratio	95% CI	*P* value
Total (*n* = 602)			
RVR	2.293	1.325–3.970	0.003
rs12979860 CC type	2.482	1.332–4.627	0.004
Viral load < 40 × 10^4^ IU/mL	2.227	1.342–3.690	0.002
Platelet ≥ 15 × 10^4^/*μ*L	1.695	1.054–2.723	0.029
Genotype 1 (*n* = 263)			
RVR	2.786	1.487–3.970	0.001
rs12979860 CC type	2.881	1.216–6.827	0.016
Genotype 2 (*n* = 297)			
Viral load <40 × 10^4^ IU/mL	3.086	1.570–6.060	0.001
RVR	2.873	1.113–7.19	0.029
Platelet ≥ 15 × 10^4^/*μ*L	2.417	1.240–4.714	0.010
N-L ratio > 1.42	0.494	0.253–0.963	0.038

CI: confidence interval; RVR: rapid virological response; N-L ratio: neutrophil-lymphocyte ratio.
